# An Account of an Uncommon Inflammation of the Epiglottis

**Published:** 1791

**Authors:** Thomas Mainwaring

**Affiliations:** Apothecary in London.


III. *An Account of an uncommon Inflammation of the Epiglottis. By Mr.* Thomas Mainwaring, *Apothecary in London. Communicated in a Letter to* Everard Home, *Eſq. F. R. S., and by him to Dr.* Simmons.

A Gentleman, about forty years of age, who had been expoſed to the influence of cold, on the 17th of December, 1790, was attacked, in the night, with a violent pain in his throat, and a total inability to ſwallow.

In the morning of the 18th the ſymptoms were a good deal increaſed. The pain was not in the ſituation uſual in ſimilar affections, but lower down, and felt more anteriorly. When he attempted to ſwallow fluids, they paſſed readily dily to the root of the tongue, where they were not allowed to remain for a moment, but were immediately forced out of the mouth with confiderable violence.

Upon examining the throat, the tonfils were in a natural ftate, as well as the palatum molle and uvula, having no tumefaction, nor were they even materially redder than common, fo that in this view of the parts there was no appearance of difeafe; but upon pulling the tongue forwards, and looking down into the throat, the epiglottis was immediately brought into view, in a very unnatural ftate, and with a very extraordinary appearance: it was much fwelled, extremely red, and looked by no means unlike the glans penis when diftended with blood in its erected ftate. It ftood directly up, fo that nothing could pafs over it, and there was very little room laterally between it and the fides of the pharynx. All the other parts were apparently free from difeafe.

A blifter was applied externally to the throat; leeches were alfo made ufe of; but there did not appear to be any abatement from either of thefe modes of treatment: the complaint continued, with little or no diminution, till the

20th,

20th, when the ſwelling, or, more properly, the ſenſibility of the epiglottis, was ſo far gone off as to allow the patient to ſwallow ſmall quantities of fluids, and by the 23d he could, with ſome pain and a little difficulty, take ſolid food.

As it is intended by the narration of this caſe principally to point out the uncommon circumſtance of an affection of the epiglottis, and that entirely independent of the other parts, the mode of treatment, particularly as it did not appear very efficacious, has been, in a great meaſure, paſſed over.

*Strand,*
February 9th, 1791.

IV. *Caſes*

